# Early Surgical Intervention in Nonbacterial Thrombotic Endocarditis to Prevent Systemic Embolization

**DOI:** 10.7759/cureus.104191

**Published:** 2026-02-24

**Authors:** Paul Weber, Dhruva Govil, Christopher Bradley, Stephen Lynch

**Affiliations:** 1 Cardiology, Henry Ford Providence Southfield Hospital, Southfield, USA; 2 Internal Medicine, Henry Ford Providence Southfield Hospital, Southfield, USA; 3 Cardiology, Henry Ford Health System, Southfield, USA

**Keywords:** aortic valve mass, echocardiogram, histopathologic evaluation, nonbacterial thrombotic endocarditis, surgical excision of mass

## Abstract

Aortic valve masses are rare and diagnostically challenging entities that may confer a significant risk of systemic embolization, particularly when large or highly mobile. We report the case of a 65-year-old woman who presented to an outside hospital with acute chest pain. Electrocardiogram demonstrated no ischemic changes, and serial cardiac troponins were negative. Transthoracic echocardiography revealed a large, mobile mass within the left ventricular outflow tract. Subsequent transesophageal echocardiography identified a 1.8-cm echogenic mass on the ventricular aspect of the aortic valve, prolapsing into the ascending aorta. An extensive infectious disease evaluation, including six sets of blood cultures, showed no evidence of infective endocarditis. Hematologic evaluation revealed a mildly elevated lupus anticoagulant, and perioperative anticoagulation with heparin was recommended, followed by rivaroxaban at discharge. Given the high embolic risk, surgical excision was pursued. Intraoperative cultures were negative, and histopathologic analysis demonstrated acellular fibrinous material. These findings were consistent with a diagnosis of nonbacterial thrombotic endocarditis (NBTE). NBTE is an uncommon condition typically associated with underlying hypercoagulable states and may closely mimic infective endocarditis in clinical and echocardiographic presentation. Early recognition of NBTE and a multidisciplinary approach involving cardiology, hematology, infectious disease, and cardiac surgery are essential to reduce embolic complications and optimize patient outcomes.

## Introduction

Intracardiac masses are uncommon but carry important diagnostic and prognostic implications when identified. Cardiac valve lesions are particularly challenging, as they span a wide differential diagnosis. Among these, aortic valve masses are especially rare, with large or highly mobile masses having a large risk of systemic embolization requiring prompt and careful evaluation. Nonbacterial thrombotic endocarditis (NBTE) is a rare and frequently underrecognized cause of valvular masses. It is characterized by sterile fibrin or platelet vegetations that form in the absence of active infection and are most often associated with hypercoagulable states, autoimmune conditions, or malignancy. The pathogenesis of NBTE involves endothelial injury, deposition of platelet-fibrin thrombi on previously normal valve surfaces, and amplification with a systemic prothrombotic state. Clinically and echocardiographically, NBTE can closely resemble infective endocarditis, often leading to diagnostic uncertainty and delays in definitive management. Distinguishing between infectious and noninfectious etiologies is critical, as treatment strategies and prognostic outcomes differ substantially.

We present a case of a large, mobile aortic valve mass initially concerning for native valve infective endocarditis (NVE) that was ultimately diagnosed as NBTE following multidisciplinary evaluation, surgical intervention, and histopathologic confirmation. This case highlights the importance of maintaining a broad differential diagnosis for valvular masses and underscores the role of coordinated, multimodal assessment in guiding timely and appropriate management.

## Case presentation

A 65-year-old woman presented to an outside hospital with acute substernal chest tightness accompanied by mild dyspnea at rest. Her medical history included hypertension, dyslipidemia, hypothyroidism, a prior transient ischemic attack four years earlier, and an unprovoked submassive pulmonary embolism two years prior, for which she was compliant with lifelong rivaroxaban therapy. She had been in her usual state of health until symptom onset the night prior to presentation, prompting emergency department evaluation the following morning.

On arrival, she was hypertensive with a blood pressure of 194/87 mmHg but otherwise hemodynamically stable, afebrile, and saturating well on room air. Electrocardiogram demonstrated a normal sinus rhythm without ischemic changes, and serial cardiac troponins were negative.

**Figure 1:**
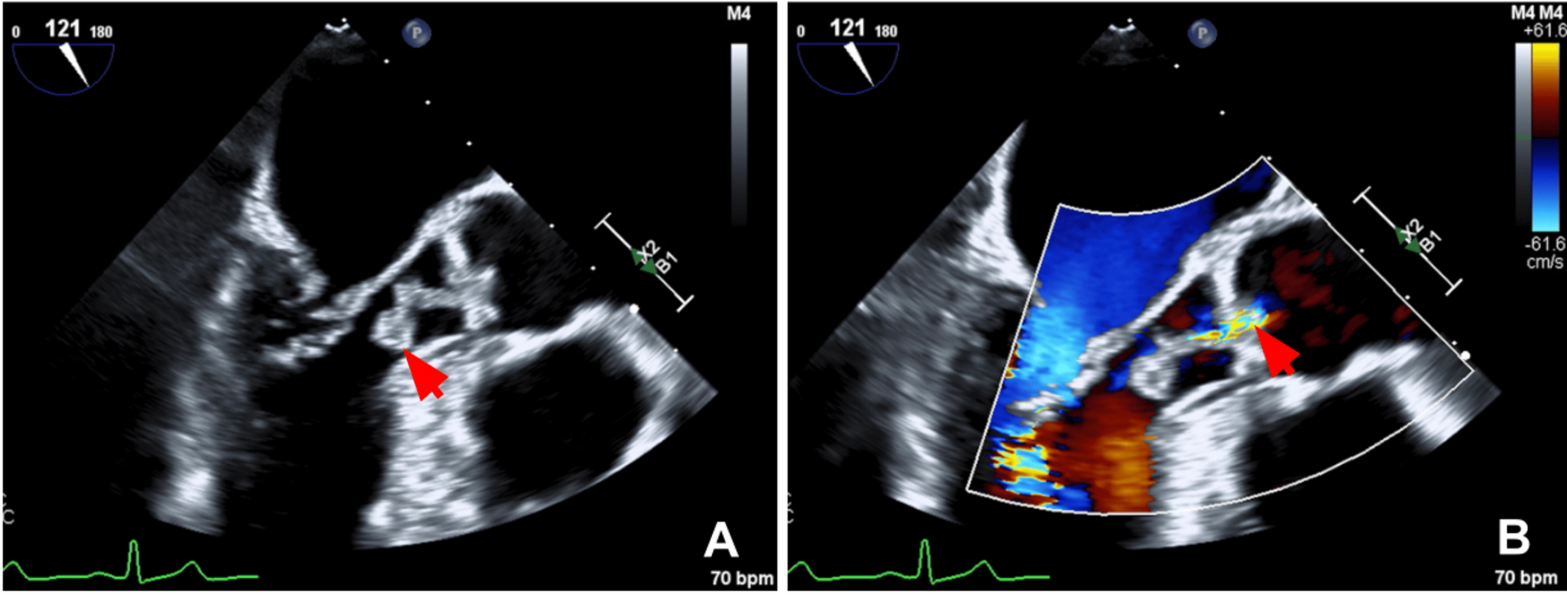
Preoperative transesophageal echocardiography of the aortic valve (A) Long-axis view demonstrating a 1.8-cm mobile mass attached to the aortic valve (red arrow). (B) Color flow Doppler imaging during diastole showing a central jet of mild aortic regurgitation (red arrow).

Transthoracic echocardiography (TTE) demonstrated a left ventricular ejection fraction of 55-60% and revealed a large, mobile mass within the left ventricular outflow tract. Transesophageal echocardiography (TEE) further characterized a 1.8-cm echogenic mass attached to the ventricular aspect of the aortic valve, prolapsing into the ascending aorta, and associated with mild aortic regurgitation (Figure 1). Laboratory evaluation revealed elevated NT-proBNP, low inflammatory markers (Table 1), and all other labs within normal limits. Given imaging findings, blood cultures were obtained.

**Table 1:** Lab results NT-proBNP: N-terminal pro-B-type natriuretic peptide

Lab	Value	Reference Range
NT-proBNP	551 pg/mL	50 - 177 pg/mL
Erythrocyte sedimentation rate	8 mm/hr	≤ 15 mm/hr
C-reactive protein	<3 mg/L	0 - 10 mg/L

Given concern for NVE, empiric broad-spectrum antibiotics and a therapeutic intravenous heparin infusion were initiated while oral anticoagulation was temporarily held. Infectious disease, cardiology, and cardiothoracic surgery consultations were obtained. The patient remained afebrile without clinical evidence of infection, and six sets of blood cultures remained negative. She did not meet the Duke criteria for infective endocarditis. Despite this, intravenous ceftriaxone was continued for presumed culture-negative endocarditis until definitive surgical pathology and intraoperative cultures excluded infection. Hematology was also consulted due to her prior unprovoked pulmonary embolism. Hypercoagulable testing revealed a mildly elevated lupus anticoagulant (50 seconds) with negative anticardiolipin, beta-2 glycoprotein, and antinuclear antibodies. There was also no concern for rivaroxaban failure, and therapeutic anticoagulation with heparin was continued perioperatively, with plans to resume rivaroxaban postoperatively.

Given the mass’s size, marked mobility, embolic risk, and lack of evidence for infection, a multidisciplinary consensus favored surgical intervention. Preoperative coronary angiography demonstrated mild, non-obstructive coronary artery disease. Cardiothoracic surgery proceeded with excision of the aortic valve mass and repair of the native valve. Intraoperative inspection via sternotomy and ascending aortotomy revealed a large mass involving all three aortic valve leaflets, which was carefully excised and submitted for culture and histopathologic analysis. Intraoperative TEE demonstrated preserved left ventricular systolic function with trace residual aortic regurgitation.

**Figure 2:**
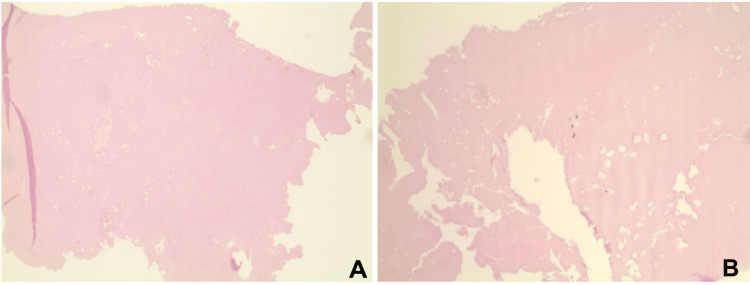
Histopathology of the aortic valve mass (A, B) Hematoxylin and eosin-stained sections demonstrate a bland, acellular eosinophilic mass composed predominantly of fibrin, without significant inflammatory infiltrate or identifiable microorganisms.

Pathologic examination of the excised mass (1.7 × 0.8 × 0.5 cm) demonstrated homogeneous eosinophilic organized fibrin without cellular elements (Figure 2). Intraoperative tissue cultures finalized after seven days showed no microbial growth, consistent with nonbacterial thrombotic endocarditis. A limited TTE on postoperative day two showed normal aortic valve structure and function with preserved ejection fraction. The postoperative course was uncomplicated, and rivaroxaban was resumed per hematology recommendations. The patient was discharged home on postoperative day three with close outpatient follow-up.

## Discussion

Cardiac valve masses, particularly those involving the aortic valve, are rare and often unexpected findings that present a significant diagnostic challenge, with an estimated incidence of less than 1 in 1,000,000 individuals. While some lesions are benign and clinically silent, others carry substantial risk, especially when large or highly mobile. The differential diagnosis is broad and includes primary cardiac tumors, thrombi, vegetations, and metastatic disease, making prompt recognition and accurate diagnosis essential. NBTE, also referred to as marantic endocarditis, is a rare, noninfectious valvular condition characterized by sterile fibrin-platelet vegetations, most commonly occurring in the setting of systemic hypercoagulability, autoimmune disease, or advanced malignancy. NBTE most frequently affects the aortic and mitral valves and is associated with a high risk of systemic embolization, approximately 40% (range 14.1%-90.9%), particularly involving the cerebral and coronary circulations [1-3].

The diagnosis of NBTE is particularly challenging, as it can closely mimic infective endocarditis both clinically and echocardiographically. Large, mobile vegetations and constitutional symptoms, particularly when accompanied by major or minor Duke clinical criteria, frequently raise concern for infectious etiology. However, features that favor NBTE include persistently negative blood cultures, normal or minimally elevated inflammatory markers, sterile tissue cultures, and pathologic findings of acellular fibrinous material [4]. In the present case, repeatedly negative blood cultures, low inflammatory markers, and sterile operative specimens shifted diagnostic suspicion away from infection and toward NBTE. Additionally, the presence of a mildly elevated lupus anticoagulant supported a transient hypercoagulable state, consistent with previously reported associations between NBTE and antiphospholipid antibody-related mechanisms [2].

Management of aortic valve masses should be individualized and guided by lesion characteristics and clinical risk, including size (≥10 mm), mobility, and embolic potential [5,6]. In NBTE, treatment primarily focuses on addressing the underlying prothrombotic condition and preventing thromboembolic complications through systemic anticoagulation. Surgical intervention is generally reserved for patients with large or highly mobile vegetations, hemodynamic compromise, recurrent embolic events, or diagnostic uncertainty [1,7]. In this case, surgical excision was appropriate given the lesion’s size and mobility, the high risk for catastrophic embolization, and the need for definitive diagnosis.

This case highlights the importance of maintaining a high index of suspicion for NBTE in patients presenting with valvular masses and persistently negative blood cultures, particularly in the absence of systemic infection or elevated inflammatory markers. Early distinction between infective and noninfective etiologies is critical, as management strategies and prognostic implications differ substantially. Multidisciplinary collaboration among cardiology, hematology, infectious disease, and cardiothoracic surgery teams was essential in establishing the diagnosis and achieving a favorable clinical outcome.

## Conclusions

Intracardiac masses remain rare but potentially high-risk findings. When located on the aortic valve, these lesions present a broad differential diagnosis that includes infective endocarditis, primary cardiac tumors, thrombi, and less common entities such as nonbacterial thrombotic endocarditis. As demonstrated in this case, distinguishing between infectious and noninfectious causes can be challenging, particularly when imaging reveals a large, mobile mass that raises immediate concern for embolic complications.

This case underscores the importance of integrating clinical presentation, microbiologic data, inflammatory markers, advanced imaging, and histopathologic analysis to establish an accurate diagnosis. Persistently negative blood cultures and low inflammatory markers should prompt consideration of alternative etiologies beyond infection. Importantly, this case supports consideration of early surgical intervention in selected high-risk patients with large, highly mobile vegetations, even before embolic events occur, when diagnostic uncertainty persists, and embolic risk is substantial. Early multidisciplinary collaboration was essential in guiding appropriate management and preventing potential catastrophic embolic events.
